# Nonlinear Time Series Analysis of Nodulation Factor Induced Calcium Oscillations: Evidence for Deterministic Chaos?

**DOI:** 10.1371/journal.pone.0006637

**Published:** 2009-08-13

**Authors:** Saul Hazledine, Jongho Sun, Derin Wysham, J. Allan Downie, Giles E. D. Oldroyd, Richard J. Morris

**Affiliations:** 1 Computational and Systems Biology, John Innes Centre, Norwich, United Kingdom; 2 Disease and Stress Biology, John Innes Centre, Norwich, United Kingdom; 3 Department of Molecular Microbiology, John Innes Centre, Norwich, United Kingdom; Harvard University, United States of America

## Abstract

Legume plants form beneficial symbiotic interactions with nitrogen fixing bacteria (called rhizobia), with the rhizobia being accommodated in unique structures on the roots of the host plant. The legume/rhizobial symbiosis is responsible for a significant proportion of the global biologically available nitrogen. The initiation of this symbiosis is governed by a characteristic calcium oscillation within the plant root hair cells and this signal is activated by the rhizobia. Recent analyses on calcium time series data have suggested that stochastic effects have a large role to play in defining the nature of the oscillations. The use of multiple nonlinear time series techniques, however, suggests an alternative interpretation, namely deterministic chaos. We provide an extensive, nonlinear time series analysis on the nature of this calcium oscillation response. We build up evidence through a series of techniques that test for determinism, quantify linear and nonlinear components, and measure the local divergence of the system. Chaos is common in nature and it seems plausible that properties of chaotic dynamics might be exploited by biological systems to control processes within the cell. Systems possessing chaotic control mechanisms are more robust in the sense that the enhanced flexibility allows more rapid response to environmental changes with less energetic costs. The desired behaviour could be most efficiently targeted in this manner, supporting some intriguing speculations about nonlinear mechanisms in biological signaling.

## Introduction

Calcium oscillations regulate a number of processes in plants, including the establishment of the legume/rhizobial symbiosis. During this interaction, bacteria (called rhizobia) invade the plant roots and are accommodated in membrane bound compartments within plant cells of a specialized organ on the root: the nodule. Within the nodule the bacteria convert atmospheric dinitrogen into ammonia, a form of nitrogen readily available to the plant. The availability of nitrogen is one of the most limiting factors for plant growth and fixed nitrogen from the legume/rhizobial symbiosis provides an essential nitrogen source for agriculture and natural ecosystems.

**Figure 1:**
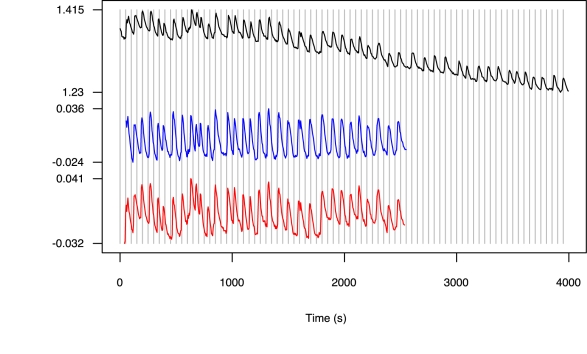
Time series Nod1 given as an example of a raw Nod Factor induced Ca^2+^ spiking trace and after detrending using a moving average (blue) and Empirical Mode Decomposition (red). The Y axis is a fluorescence ratio between Ca^2+^ sensitive and Ca^2+^ insensitive dyes. The X axis is time in seconds.

The establishment of the legume/rhizobial symbiosis involves a molecular communication between the plant and the bacteria, with bacterially-derived Nod (nodulation) factor acting as a central signal to the plant. Perception of Nod factor by legumes activates most of the developmental processes associated with the formation of a nodule. The Nod factor signal transduction pathway of legumes has been well characterized and involves calcium oscillations, termed calcium spiking. An example of calcium spiking is given in Figure 1. Receptor-like kinases are involved in the perception of Nod factor and this leads to induction of calcium spiking via cation channels, that appear to regulate potassium movement and components of the nuclear-pore complex [1]. This signal transduction pathway has also been shown to function in the establishment of a second symbiotic interaction: the mycorrhizal symbiosis. This interaction involves the colonization of the plant root by mycorrhizal fungi that aid the plant in the uptake of nutrients from the soil. Mycorrhizal fungi have been shown to activate calcium oscillations, but with a different structure to Nod factor induced calcium spiking [2]. This suggests that the symbiosis signaling pathway can be differentially activated by both rhizobia and mycorrhizal fungi.

The nature of biological systems and the challenges inherent in experimentation often result in seemingly erratic time-series behaviour with little apparent structure. Despite advances in signal processing methodology, the extraction of information from such data remains a challenge. Erratic behaviour is often thought to be the consequence of noise or stochastic effects, but apparent randomness can also be generated by a deterministic system operating in the chaotic regime. A universally accepted definition of chaos is still outstanding, however, a number of key features are held in common: A chaotic system is deterministic, nonlinear, and highly sensitive to the initial conditions. The exponential divergence of nearby trajectories implies that the predictability is limited to short time scales. Long-term forecasts become impossible despite the underlying deterministic nature. Unpredictable systems are frequently handled with the methods of probability theory and termed stochastic.

Sophisticated techniques exist for distinguishing between linear, nonlinear, deterministic, stochastic and chaotic systems. However, disentangling experimental noise, stochastic effects, and underlying deterministic laws is non-trivial and the initial data derived from biological processes are not often of sufficient quality to allow such analyses. Experimental investigations into calcium (Ca^2+^) oscillations have frequently been accompanied by mathematical modeling and a wide range of models exist (see [3] for an excellent review of this topic). Questions, however, remain as the mechanisms responsible for the Ca^2+^ signal en- and decoding likely vary between organisms and are not fully understood.

For example, intracellular Ca^2+^ oscillations and Ca^2+^ spikes have been modeled with chaotic systems [4], [5], [6], although stochastic descriptions have been proposed for some of the ion channels involved [7]. Initial chaotic models were inspired by the bursting behaviour observed in experiments on hepatocytes [8], [9], [10]. However, a later theoretical study has shown that an example Ca^2+^ oscillatory system can only be modeled deterministically at physiological Ca^2+^ concentrations when bursting is not taking place [11].

A recent study on Ca^2+^ oscillation data from hepatocytes, which included bursting, led to the conclusion that calcium oscillations were predominately stochastic in nature [12]. Time series data from four cell types in mice and humans was used to show a rapidly falling autocorrelation between Ca^2+^ spike intervals [13]. This was interpreted as evidence that Ca^2+^ spikes are initiated stochastically. Further analysis revealed that the statistics of the interspike intervals are in agreement with a stochastic model.

In plants, moreover, little is known about the secondary messengers or calcium channels that may direct Nod factor induced calcium spiking [14], also it is apparent that there are major differences in the proteins that activate or perceive well-characterized animal secondary messengers such as inositol trisphosphate and cADPR [15]. Given these unknowns and differences, we are reluctant to bias our analysis towards the models and conclusions drawn from animal systems. Instead, a more appropriate approach to understand Nod factor signaling is to analyse the experimentally obtained calcium oscillations using methods from nonlinear time series analysis. Using a series of techniques, we demonstrate that Nod factor induced Ca^2+^ oscillations generated within the legume *M. truncatula* are deterministic, nonlinear and show an exponential divergence that is typical of chaotic systems. This observation suggested an alternative explanation to a stochastic interpretation and prompted us to validate our methodology using negative and positive controls. We generated time series using the chaotic Lorenz system of differential equations and the chaotic Haberichter model of Ca^2+^ oscillations. These models were tested alongside our experimental data. We find that while both these positive control data sets would be classified as chaotic using many classical methods, they would be categorized as stochastic using the methods employed in recently published time series analyses of Ca^2+^ oscillations. Whereas stochastic modeling is often an effective approach, the extrapolation from a modeling convenience to the nature of observed phenomena is not without risk and interesting phenomena may be overseen and/or ascribed to random effects. We therefore take a number of precautions to present as thorough an analysis as possible of the experimental Ca^2+^ oscillations.

## Results

**Figure 2:**
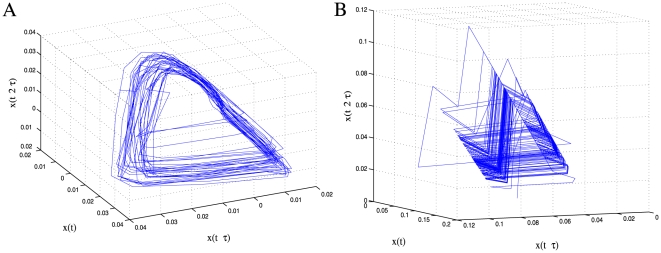
Three dimensional embeddings of a time series of fluorescence ratios (Nod1) and the stochastic spiking model data for comparison. A: The experimental series is clearly noisy, prohibiting accurate dimensionality determination, but it unfolds well to the eye in three dimensions. B: The data from the stochastic spiking model, however, appears to cross itself in many places and coalesces, violating the uniqueness property of ODE.

In the following we describe the results of a number of nonlinear time series analyses. In order to check whether a stream of data has arisen from a chaotic system, a number of tests must be carried out. Definitive answers are rare unless the system of underlying equations or map is known. Plotting system observables as a function of themselves at an earlier time gives rise to the return map, which often appears as a simple curve for deterministic systems. The shape of such a curve strongly indicates the classification of the dynamics. This technique is in fact a form of state space reconstruction, in which typical deterministic trajectories should establish themselves upon a low-dimensional attractor. A further test is for exponential divergence and the calculation of Lyapunov exponents, which if positive indicates chaos. These tests are sensitive to noise, which is always present especially in biological data, and hence rarely provide definitive answers. One of the key steps for such analyses is proper embedding and the determination of attractor dimensionality. Current approaches for these steps work well for data with in the order of 2% noise but perform unreliably for noisy data sets. Thus, we are limited in the application of such methods and as a result could not determine the dimensionality reliably, and the return map computations did not produce convincing results. However, as can be seen in Figure 2, the attractor does appear to unfold well in three dimensions. Additionally, a number of tests did provide useful results with a good confidence level. The following sections describe the application of a number of different tests, which taken together certainly do not prove but provide evidence for chaos.

The results are described in two sections. The first section provides accumulated evidence for chaotic behaviour in the Ca^2+^ time series in *M. truncatula*. In the second section, additional tests allow a comparison to previous time series analyses that were performed on Ca^2+^ oscillations in animals.

### Evidence of Chaos?

**Figure 3:**
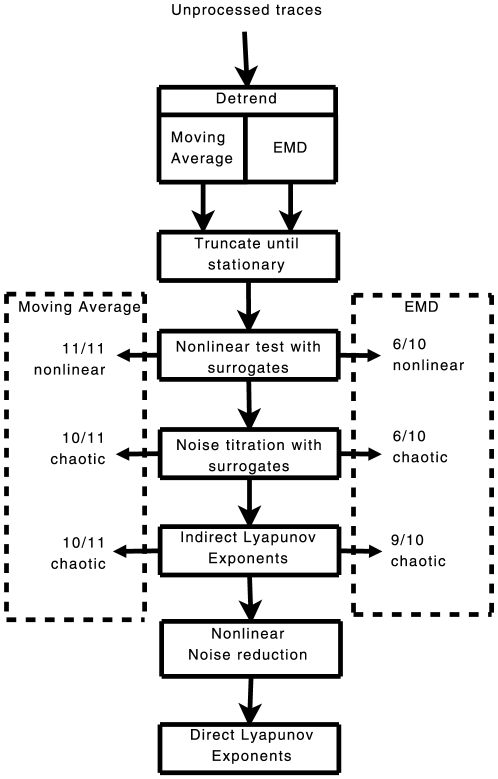
Flowchart of the tests run to gather evidence for chaos. A summary of results on the left of the figure are after processing with a moving average. The summary of results on the right are after detrending with Empirical Mode Decomposition (EMD).

We analysed the Ca^2+^ oscillations (Supporting Information Experimental Data S1) by following the procedure illustrated in the flowchart of Figure 3. The full time series are used and not just interspike times. The time series of Ca^2+^ concentration were first detrended using two different techniques, Empirical Mode Decomposition (EMD) and a moving average (Figures S1, S2, S3, S4, S5, S6, S7, S8, S9). Using EMD does not distort the shape of the Ca^2+^ spikes and does not remove low frequency components of the experimental signal. However, because the low frequency components of the signal may not be significant, as an alternative to EMD we also detrended the data using a moving average.

Each detrended Ca^2+^ spiking time series was tested for nonlinearity using a nonlinear predictor and linear surrogates. If nonlinearity was detected, a noise titration was used to test for chaos and the Lyapunov exponent was calculated using a direct method. The direct method calculates the maximal Lyapunov exponent and inspection of the resulting divergence data can help one to discern if the divergence of the system is due to chaotic or stochastic effects. An indirect method was also used where multiple nonlinear models were fitted to the experimental data and a maximal exponent calculated for each model. The indirect method gave a selection of Lyapunov exponents and if a clear majority of well fitting models had positive exponents then we take this as evidence that the divergence is more likely due to deterministic chaos rather than stochasticity (Figure S12).

### Nonlinearity, Noise Titration and Lyapunov Exponents

Evidence of chaos was suggested in the majority of traces (16 out of 21) using a noise titration with the surrogates nonlinear test (Table 1). Applied to linear autoregressive (AR) models fitted to the experimental data, this test correctly identified forty true negatives and only two false positives. In some cases, the results of the experimental time series vary depending on the method of detrending, with some (4 out of 10) EMD detrended time series failing the test for nonlinearity.

**Table 1:** Stationary time series of Ca concentration with lengths given by the number of samples.

Time Series	Detrending	Length	# Spikes	Zeroth	Noise Titration%
Nod1	MA	500	31	0.01	16
	EMD	500	34	0.01	16
Nod2	MA	400	36	0.01	25
Nod3	MA	700	45	0.01	23
Nod4	EMD	600	46	0.02	20
Nod5	MA	480	30	0.01	20
	EMD	741	46	0.01	12
Nod6	MA	339	14	0.01	0
	EMD	359	15	0.20	0
Nod7	MA	1058	46	0.01	9
	EMD	1170	50	0.01	11
Nod8	MA	1100	55	0.01	15
	EMD	1029	47	0.82	0
Nod9	MA	409	15	0.01	1
	EMD	260	9	0.83	0
Nod10	MA	400	22	0.01	20
	EMD	520	30	0.01	16
Nod11	MA	440	46	0.04	24
	EMD	700	67	0.02	13
Nod12	MA	600	34	0.01	23
	EMD	760	43	0.33	0
Stochastic interspike	-	1000	73	0.01	34

The sample time is 5 seconds. The spikes column indicates the number of spikes in the time series. P-values are given for the null hypothesis that each nod factor time series was generated by a linear process. The *p*-values are calculated by running the zeroth surrogates test, which was also used for a noise titration to get a limit for the noise that could be added without destroying evidence of nonlinearity.

**Figure 4:**
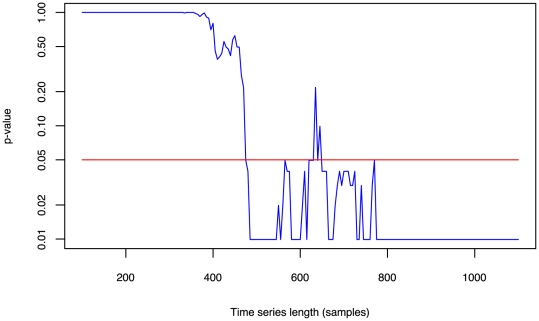
Semi-log plot of *p*-value against series length for a single time series that passes the zeroth surrogates test for nonlinearity. Each *p*-value was calculated using 100 surrogates. Signs of nonlinearity are not detected until the length of series being tested is greater than 400 samples. P-values do not drop to show significant nonlinearity, which is marked with a red line, until the time series is approximately 500 samples long.

The nonlinear surrogates test exhibits a length dependence and so the shorter time series failed (Table 1). The nonlinear predictability was computed for a long time series that was steadily truncated to provide a comparison of *p*-value against series length (Figure 4). The *p*-values do not consistently indicate nonlinearity for times shorter than 400 samples.

An indirect method for maximal Lyapunov exponent calculation that fitted deterministic models to the Ca^2+^ time series, gave positive exponents for all experimental time series except for Nod3 and Nod4. All negative controls correctly gave negative maximal Lyapunov exponents.

**Figure 5:**
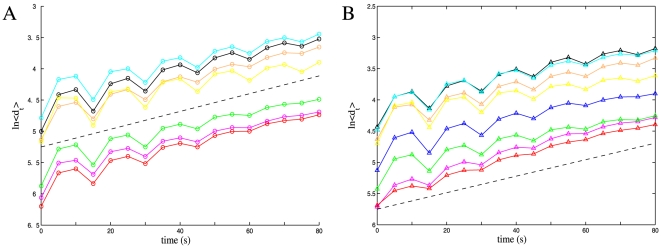
Semi-log plot of the average divergence <d_t_> of the nearest neighbors of each point in the time series as a function of time. The short term fluctuations are due to the periodicity of the signal, but the average distance clearly grows exponentially. The data points pictured are for an embedding dimension of seven, and consistent values for the maximal exponent are achieved once the dimension is greater than or equal to six. The exponent is 0.0142 for traces detrended with a moving average (A), and 0.0132 for EMD (B). These values are given by the slope of the black dashed lines. For each trace, the exponent was computed as the average of three slopes: 1) the slope through local maxima; 2) the slope through local minima; and 3) the slope through the average of local minima and maxima. The final value of the exponent was computed by averaging over all traces. Computations were not done for traces Nod6 and Nod9 for either detrending because of the short series length. We remark that the same computations for the stochastic spiking model give a semi-log plot of the form <d_t_> ∼ t^1/A^, indicating diffusive-like divergence.

Since the majority of the traces passed a test for nonlinearity the system can be considered nonlinear, justifying the application of nonlinear noise reduction techniques. Once the experimental Ca^2+^ spiking traces were noise-reduced, a direct Lyapunov calculation method was performed. The logarithm of the divergence of neighbouring points in phase space against time revealed a clear linear trend in the majority of the time series, indicating exponential divergence. This is shown in Figure 5. Taking an average gradient gave a Lyapunov exponent of 0.014 s^−1^ for time series detrended using a moving average and 0.013 s^−1^ for time series detrended using EMD.

### Tests for comparison

#### Nonlinear System with Random Interspike Intervals

**Figure 6:**
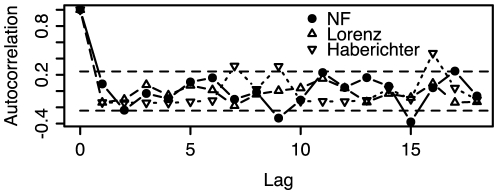
Autocorrelation of cycle times for a Nod Factor time series and two positive controls based on chaotic mathematical models. The X axis is the lag measured in number of samples (sample time is 5 seconds). All time series show a rapid drop to within the 95% confidence interval for white noise which is marked with horizontal dashed lines and represents no identifiably repeating patterns.

Properties of the autocorrelation of interspike intervals have been used to support the idea of stochastic spike activation in four cell types from mice and humans [13]. In order to compare our initial results, which tend to support the case for determinism, with the stochastic hypothesis, the autocorrelation of the intervals between maxima was calculated for two known chaotic differential equations and an experimental Nod factor Ca^2+^ spiking time series. For a purely random time series (white noise) the autocorrelation is close to zero. This is depicted in Figure 6 in which horizontal dashed lines mark the approximate 95% confidence interval for white noise. This confidence interval is calculated as ±1.96/√*N*, where *N* is the length of the series of interspike intervals. Both the mathematical models and the experimental data show a rapid drop in autocorrelation indicating that successive intervals are not correlated. However, the mathematical models act as positive controls revealing that the drop in autocorrelation is not necessarily down to stochastic effects.

It must be pointed out that nonlinear time series analyses cannot provide a definite answer regarding the nature of spike activation and interspike times in the system. We considered a nonlinear deterministic model for the spike waveforms, with randomly chosen interspike intervals. As expected, this signal clearly appears nonlinear; however, it also appears chaotic using a noise titration. This demonstrates that some conventional tests used to detect chaos are unable to discern between purely chaotic systems and a carefully designed deterministic spiking system with stochastic activation. For this reason we use a number of different techniques with the goal of presenting as thorough an analysis of the experimental Ca^2+^ oscillations as possible. A direct Lyapunov calculation for the time series with stochastic interspike times does not exhibit a clear exponential divergence. Figure 5 shows the divergence to be of the form t^1/α^, characteristic of a randomly perturbed deterministic system. The indirect method also indicates that the majority of models fitted to the time series with stochastic interspike intervals have a negative Lyapunov exponent.

### Determinism

The results of determinism tests are somewhat subjective and were therefore not used to support our conclusions. In contrast, the findings from one such test have been used as evidence of stochasticity in Ca^2+^ oscillations [12]. All traces obtained from our experiments pass the three statistical tests for determinism proposed by Aparicio [16] without the use of noise reduction. A negative control using random numbers fails the three determinism tests.

We evaluated the Kaplan and Glass measure for determinism on the Lorenz system and the Haberichter chaotic model of Ca^2+^ oscillations, both with 10% noise added to mimic the noise estimated in the experimental data. This method is based on a vector reconstruction of the attractor over a grid of 5^6^ boxes. It gives a determinism factor, −Λ, of −Λ = 1 for full determinism and −Λ = 0 indicates complete randomness. The Lorenz time series had a determinism factor of −Λ = 0.88, and −Λ = 0.78 for the chaotic spiking model. Both of these time series would be classed stochastic using the criteria from other studies which required −Λ>0.9. More than highlighting deficiencies of the Kaplan Glass test, these results show the limitations in using only one metric to characterize noisy data sets.

## Discussion

Chaos is common in nature. For instance, the gravitational three body problem can exhibit deterministic chaos and numerous further examples exist for which chaotic behaviour have been identified or suggested, ranging from the solar system, weather, population dynamics, to Brownian motion and diffusion [17]. An interesting example of the potential relevance of chaotic flexibility has been discussed for human heart beats. It has been suggested that normal heart behaviour might be chaotic and can thus respond efficiently to perturbed conditions, whereas diseased hearts are more stable in their frequencies and less able to make necessary adjustments to stress [18]. However, chaos may also be involved in the destabilisation of heart rhythms, as quasiperiodicity and intermittency have been observed in the Ca^2+^ oscillations of cultured cardiomyocytes degenerating into chaos-like behaviour that would be fatal *in-vivo* [19]. Whether or not biological systems such as the heart or brain are really chaotic is still the subject of much debate and on-going research.

Using a range of techniques from nonlinear time series analysis we have gained some evidence suggesting that the Ca^2+^ spiking in the root hair cells of *M. truncatula* might be chaotic. We first demonstrated that the majority of the time series show the Ca^2+^ oscillations to be nonlinear. To check for false positives we also tested linear models fit to the experimental data. The two false positives we obtained show that the test for nonlinearity can be fallible in some cases, should not be considered absolute, but nevertheless provides evidence of nonlinearity.

We then performed a test for chaos using the noise titration technique. This test indicated that the majority of the Ca^2+^ time series were nonlinear in the presence of additive noise. This can be viewed as evidence of chaos [20]. Although the majority (19 out of 21) of the negative controls were correctly identified, the two false positives from the nonlinear test also passed the noise titration. Furthermore, a synthetically produced time series consisting of deterministic spikes separated by stochastic interspike intervals - a model that has been proposed for Ca^2+^ oscillations in animal systems - was also classed as chaotic by the noise titration method. Although this model is largely deterministic and nonlinear, it is not chaotic. This demonstrates that classification using the noise titration method should be done with caution.

Using a combination of an indirect method to compute the probable sign of Lyapunov exponents and a direct method to calculate the magnitude and type of the divergence, evidence of chaos was revealed in the Ca^2+^ oscillations and controls without any false positives. To our knowledge, this particular combination of direct and indirect methods of Lyapunov exponent calculation with the use of controls has not been used before.

In animals, the hypothesis that Ca^2+^ oscillations, experimentally obtained from hepatocytes, originated from a deterministic system was rejected [12]. The conclusion that these oscillations are “prevalently stochastic” was reached because one of two time series failed a nonlinear test, and the one that passed had a determinism score of −Λ <0.9 as provided by a Kaplan-Glass analysis. We have given two examples of chaotic oscillations that fail to meet this criterian under similar noise conditions to the experimental data being considered. The noise present in our experimental data (around 10%), results in some of the individual tests producing inconclusive answers, but the combination of all results presents a stronger case which suggests that the oscillations in *M. truncatula* are produced by a nonlinear, deterministic system.

In order to understand the fundamental nature of seemingly erratic calcium oscillations, the question of randomness or chaos arises and needs to be sufficiently addressed. To indisputably demonstrate stochasticity as a main driving mechanism in calcium oscillations, determinism must be eliminated. This is a non-trivial task for a number of reasons. Fundamentally, given that noise is nearly always present and the high demand on data quality and quantity for most non-linear techniques to work robustly, this distinction between stochasticity, noise, and low-dimensional chaos can rarely be achieved. Practically, the choice of parameterisation is often known to be approximate and deviations therefrom are called stochastic effects within the chosen framework and reduced phase space. However, there now exists a wealth of advanced tools and approaches from time series analyses and dynamical systems theory, which can be employed to shed light on the nature of experimental data and offer possible interpretations. In accepting randomness too readily, the exciting discovery of a biological system taking advantage of attributes of chaotic motion would be missed and some of its most interesting features labeled as chance occurrences.

A number of properties of dissipative chaotic systems make them suitable for Ca^2+^ signaling. First, and perhaps counterintuitively, a theoretical study on Ca^2+^ oscillations has shown that both the sensitivity to parameter perturbations and the capacity to attune to a forcing frequency do not depend on the oscillations being chaotic or regular [21]. This means that, despite the sensitive dependence on initial conditions, chaotic systems can be equally robust and flexible as regular systems in a highly variable biological environment. While these statements are based upon evidence from a particular model, they can be generalized.

In non-conservative systems, chaotic trajectories are restricted to lie upon either strange attractors or chaotic saddles. These two cases represent sustained or transient chaos, respectively. The saddles are hyperbolic, and as such they are structurally stable and deform smoothly with parameters. Moreover, it has been shown that the transient time spent tracing a chaotic saddle changes slowly with increasing levels of noise [22]. The case of sustained chaos is similar: strange attractors typically retain their shape regardless of small parameter perturbation (except at crisis values). Thus the trajectories that trace the attractors also maintain their characteristic shape in noisy environments. The consequence is that the patterns made by system observables – here the oscillating Ca^2+^ level – can be robust despite fluctuations.

These qualities are advantageous to the symbiosis signaling pathway under study, which has been shown to take part in two important symbioses that are evolutionary separated by hundreds of millions of years [2]. Each symbiosis leads to a different Ca^2+^ oscillation signature despite the use of common components. The existence of the multiple steady states excludes the possibility that the signaling pathway is a stationary, linear process.

The possibility that the system jumps from one attractor to another in response to different input signals would have important implications, but the capacity for dual signal generation could also be a sign that the system is controlling chaos, i.e. the two signals represent subregions of a larger chaotic set. The control of chaotic motion, as originally proposed [23], utilizes that the state of the system visits any neighborhood of periodic orbits of every period. Tiny controlling effects can then be adeptly used to direct the behaviour to any periodic motion. The concept has been widely used in circuitry, lasers, chemistry, low-energy orbit design, and even to direct the rhythms of the heart. In the case of Ca^2+^ oscillations one candidate for the source of the pertubations is Ca^2+^ influx [24]. The control algorithm is attractive because of its efficiency, and could be used here to maintain the periodicity of the oscillations, to synchronize spatially separate components, or to specify one of the two signals.

It remains to be discovered whether chaos control is being harnessed for the efficient tuning of the Ca^2+^ oscillations or if chaotic flexibility is an essential factor for signaling specificity. Discovering further examples of nonlinearities and chaos within the cell would have implications for the way we view the principles of signaling pathways. One reason to suspect intracellular chaos is simply that it can be produced rather easily by relatively few strongly-interacting components, and it is common in many natural systems. As has been shown for noise, biological systems are capable of using common effects to its advantage. Given that chaotic systems can indeed be robust, and that chaos control enhances adaptability to environmental changes at less energetic costs and with accurate targeting of desired behaviour, we find this a fascinating speculation for biological signaling. It may come as no surprise to learn that evolution could have beaten physicists to the discovery that small perturbations can be efficiently used to control chaotic systems [25], [26].

## Materials and Methods

In the following, we concisely explain the methods employed in this study. For readers who are not familiar with nonlinear time series analysis, we provide additional background in Supporting Information Additional Background S1.

### Time Series and Controls

We analysed time series data obtained from root hair cells of *M. truncatula* treated with M Nod factor from *S. meliloti*. The nature of the Ca^2+^ oscillations is comparable whether the plant is treated directly with Nod factor or with *S. meliloti* [27], but for ease of experimentation in this study we have chosen to use isolated Nod factor. The changes in Ca^2+^ levels were measured using the ratio of fluorescence from two dyes: Oregon Green that responds to calcium levels with changes in its fluorescence and Texas Red that is not responsive to calcium and provides a control for fluorescence changes unrelated to calcium. These dyes were micro-injected into root hair cells and fluorescence measured as described in [28]. The intensity of the fluorescence was measured in individual cells at five second intervals for a period of at least 60 minutes. Examples of an unprocessed time series and detrended time series are given in Figure 1.

Experimental time series from 9 cells, were analysed. After detrending by two methods, splitting some time series according to stationarity tests and removing one EMD detrended time series due to nonstationarity, we were left with a total of 21 Ca^2+^ spiking time series.

The comparison of the autocorrelation of interspike intervals used two time series obtained from chaotic mathematical models as positive controls. One of the positive controls was generated by a model of Ca^2+^ spiking developed by Haberichter *et al* [6] and the other by the well known chaotic Lorenz system [29]. Tests for determinism used a time series generated with random numbers obtained from http://www.randomnumbers.info/ as a negative control. As negative controls for nonlinearity we produced two time series, an instance of an autoregressive (AR) model, and a surrogate [30], from each experimental time series (Figure S10). To see the effects of a time series analysis on the type of system suggested by [13], a simple nonlinear model with random interspike intervals was tested.

### Random Interspike Intervals

The following model was used to generate a synthetic time series as a negative control. The model has a state *S*∈{*spike*,*release*}, the time since the last spike, τ, total spikes *n*, and a set of interspike intervals that follow a normal distribution, {α}∼ N(μ,σ):
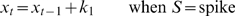
(1)

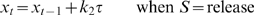
(2)


The model produces a linear spike followed by an exponential decay. The state changes from *spike* to *release* when *x_t_* exceeds a threshold value. Stochasticity is introduced by changing the state from *release* to *spike* when τ = α*_i_*. The shape of the spikes are controlled by the constants *k_1_* and *k_2_*.

### Detrending

Cytoplasmic streaming causes relocalisation of the fluorescent dye and this coupled to photobleaching causes noticeable Ca^2+^ independent changes in the overall fluorescence. The ratio of the Ca^2+^ responsive dye, Oregon green, to the non responsive dye, Texas red, reduces some of these non-specific fluctuations, but does not remove all Ca^2+^ independent changes in fluorescence. To remove these effects a moving average was taken and the result subtracted from the time series [31]. The number of points in the moving average was particular to each trace and was set to either 19 or 25 points. Changing the number of points gave control over the type of features to be removed.

The moving average is a linear method and can obscure non-linearities within the signal. A further danger arises in that human judgment of how many points to include in the moving average may affect the final results. Because of this potential for bias, the Nod factor induced spiking time series were also separately detrended by Empirical Mode Decomposition (EMD) [32]. This method of detrending deals more formally with the nonlinearity of the time series and does not distort the shape of the Ca^2+^ spikes [33], however like most automatic methods it is unable to apply heuristic information and could fail to remove experimental idiosyncrasies.

### Stationarity

In order to detect possible parameter changes, such as alterations in temperature, which could affect the period and shape of the Ca^2+^ oscillations, a test for non-stationarity [34] was run on each time series. Whenever there was evidence of a parameter change, given by a cluster of *p*-values below 0.05 along a section of the time series, the series was cropped before the section showing the parameter change. The detrended time series in Figure 1 were truncated in this way. The complete and truncated time series are shown in the Supporting Information (Figure S11).

### Time Delay Embedding

The percentage of false nearest neighbours was graphed for embedding dimension, *m*, where 2≤*m*≤10. The embedding dimension *m* = 6 was chosen after reviewing all the traces for a dip in the percentage of false nearest neighbours. This embedding would be suitable for an attractor which has up to three dimensions [35].

A delay time of fifteen seconds for the embedding was suggested by three different methods: mutual information [35], a drop in autocorrelation to (1−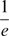) [36] and considering time window length [37].

### Detecting Determinism

Recurrence quantification analysis [16] was run on the detrended time series to detect determinism. The Kaplan Glass test for determinism [38], [39] was used on a grid of size five with a six dimensional embedding to plot the average box vector length, −*L*_n_, against the number of passes, *n*, and to calculate a determinism factor −Λ.

### Noise Reduction

When working with Ca^2+^ spiking traces, we found a straightforward noise reduction method [40] was able to remove the background activity and preserve the spikes in agreement with qualitative inspection from experimentalists. The neighbourhood size was chosen by inspecting autocorrelation plots of the removed noise and cross-correlation plots of the noise against remaining signal. We chose the largest neighbourhood size that combined rapidly decaying autocorrelation with insignificant cross-correlation.

### Testing for Nonlinearity

The performance of a nonlinear predictor on experimental data can be used to test for nonlinearity. These results were compared against those obtained from numerically generated surrogate time series [41]. This comparison resulted in a p-value for the null hypothesis that the Ca^2+^ spiking time series is linear. Following [41], if the p-value<0.05 the experimental time series was classed as nonlinear and a noise titration was performed.

### Noise Titration

Nonlinear tests can be used to detect chaos using a noise titration technique [20]. The titration was used in conjunction with the nonlinear test that utilised surrogates, as we found this test more noise resistant than the test conventionally used with a noise titration. Similiar observations have been reported by [41].

### Direct Method for Maximal Lyapunov Exponents

The maximal Lyapunov exponent was calculated using a method proposed by Rosenstein [36] suited to short time series of lengths in the order of 1000 points.

### Indirect Method for Maximal Lyapunov Exponents

The indirect method fitted many nonlinear models to each time series. Each model had the form,
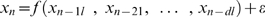
where *f* is a function produced by fitting a neural network [42] to the time series under study, *d* is the embedding dimension, *l* the time delay and *ε* is a noise term. Multiple models were fitted for each value of *d* and *l*.

A Lyapunov exponent was then calculated from each fitted model along with a Bayesian Information Criterion (BIC) to describe how well each model fitted the data under test. The sign of the Lyapunov exponent was determined by the sign of the exponents for a majority of the fitted models with a lower BIC.

### Computational Tools

Time series analysis was carried out with the Tisean package [43], version 3.01, using the RTisean interface for the *R* statistical environment [44] unless stated otherwise. The Empirical Mode Decomposition was performed by a program provided by Patrick Flandrin at ENS Lyon [45]. AR models were generated in R using the Burg algorithm. The Kennel test of nonstationarity and determinism tests were developed using R. The Lyapunov exponents were computed directly using code provided by Rosenstein [36]. The indirect Lyapunov exponents were calculated using the LENNS program version 1.0 [42].

## Supporting Information

Experimental Data S1 The experimental calcium traces
(0.10 MB ZIP)

Additional Background S1 We provide additional theoretical background information on the computational methods as most readers may not be familiar with the employed techniques. There is no one good source for these methods and interested readers would therefore have to consult a number of books and papers to gather this information.
(0.05 MB TAR)

Figure S1 The Nod1 time series (black), after detrending with a moving average (blue) and after detrending using EMD (red). Y axis is a fluorescence ratio between Ca^2+^ sensitive and Ca^2+^ insensitive dyes. The detrended time series have been truncated to make stationary.
(0.12 MB EPS)

Figure S2 An original experimental time series (red), after detrending with a moving average to produce Nod2 (green) and Nod3 (cyan) and after detrending using EMD to produce Nod4 (purple). Y axis is a fluorescence ratio between Ca^2+^ sensitive and Ca^2+^ insensitive dyes. The detrended time series were truncated to obtain long, stationary segments.
(0.23 MB EPS)

Figure S3 The Nod5 time series (black), after detrending with a moving average (blue) and after detrending using EMD (red). Y axis is a fluorescence ratio between Ca^2+^ sensitive and Ca^2+^ insensitive dyes. The detrended time series were truncated to obtain long, stationary segments.
(0.14 MB EPS)

Figure S4 The Nod6 time series (black), after detrending with a moving average (blue) and after detrending using EMD (red). Y axis is a fluorescence ratio between Ca^2+^ sensitive and Ca^2+^ insensitive dyes. The detrended time series were truncated to obtain long, stationary segments.
(0.11 MB EPS)

Figure S5 The Nod7 time series (black), after detrending with a moving average (blue) and after detrending using EMD (red). Y axis is a fluorescence ratio between Ca^2+^ sensitive and Ca^2+^ insensitive dyes. The detrended time series were truncated to obtain long, stationary segments.
(0.20 MB EPS)

Figure S6 The Nod8 time series (black), after detrending with a moving average (blue) and after detrending using EMD (red). Y axis is a fluorescence ratio between Ca^2+^ sensitive and Ca^2+^ insensitive dyes. The detrended time series were truncated to obtain long, stationary segments.
(0.21 MB EPS)

Figure S7 The Nod9 time series (black), after detrending with a moving average (blue) and after detrending using EMD (red). Y axis is a fluorescence ratio between Ca^2+^ sensitive and Ca^2+^ insensitive dyes. The detrended time series were truncated to obtain long, stationary segments.
(0.11 MB EPS)

Figure S8 The Nod10 time series (black), after detrending with a moving average (blue) and after detrending using EMD (red). Y axis is a fluorescence ratio between Ca^2+^ sensitive and Ca^2+^ insensitive dyes. The detrended time series were truncated to obtain long, stationary segments.
(0.18 MB EPS)

Figure S9 An original experimental time series (black), after detrending with a mov- ing average to produce Nod11 (blue) and Nod12 (cyan) and after detrending using EMD to produce Nod11 (red) and Nod12 (purple). Y axis is a fluorescence ratio between Ca^2+^ sensitive and Ca^2+^ insensitive dyes. The detrended time series were truncated to obtain long, stationary segments.
(0.25 MB EPS)

Figure S10 Examples of an experimental time series after moving average detrending (red), an AR model fitted to the experimental data (green) and a surrogate time series (blue). Y axis is a fluorescence ratio between Ca^2+^ sensitive and Ca^2+^ insensitive dyes.
(0.14 MB EPS)

Figure S11 Example of a time series truncated for stationarity. a) The original time series is given in red and the time series, after trunctation, is given in blue. b) The cluster of p -values (y axis) that indicate nonstationarity when the entire series is analysed. c) The results of the staionarity test after truncation. The red line marks the p -value 0.05 which is used as a cutoff for clusters of non-stationary p -values. When a a p -value is calculated that is <0.05 it is marked with a red dot.
(0.29 MB EPS)

Figure S12 Examples of indirect Lyapunov exponents calculated for an experimental time series and an AR model used as a negative control. The ‘L’ values indicate the delay time or lag that was used for particular models and the points are only plotted for the best fitting model for each dimension and lag.
(0.22 MB EPS)
